# Validation of the International Academy of Cytology Yokohama System for Reporting Breast Fine-Needle Aspiration Cytopathology

**DOI:** 10.7759/cureus.113225

**Published:** 2026-07-23

**Authors:** Shyamala Gowri, Banushree CS, Valli Priyaa, Simon D Dasiah

**Affiliations:** 1 Pathology, Aarupadai Veedu Medical College and Hospital, Vinayaka Mission's Research Foundation (Deemed to be University), Puducherry, IND; 2 Pathology, Indira Gandhi Medical College and Research Institute, Puducherry, IND; 3 Pathology, All India Institute of Medical Sciences, Madurai, Madurai, IND; 4 General Surgery, Indira Gandhi Medical College and Research Institute, Puducherry, IND

**Keywords:** accuracy, breast lesions, fine needle aspiration cytology, iac, risk of malignancy, yokohama system

## Abstract

Background

The International Academy of Cytology (IAC) Yokohama System for the classification of fine-needle aspiration cytology (FNAC) is a diagnostic tool for breast lumps. Cyto-histopathological correlation increases diagnostic precision and is of great relevance.

Aim

To classify the FNAC of breast lesions according to the IAC Yokohama classification system and to study sensitivity, specificity, and risk of malignancy (ROM) by correlating with histopathological examination.

Settings and design

A three-year retrospective study of 63 breast FNAC cases with histopathological correlation was performed using the Yokohama System.

Materials and methods

Taking histopathological diagnosis as the gold standard, diagnostic indices, including sensitivity, specificity, positive predictive value (PPV), and negative predictive value (NPV), were evaluated. The 63 cases were subsequently recategorized according to the Yokohama System into the following categories: insufficient, benign, atypical, suspicious for malignancy, and malignant.

Results

The ROM across the Yokohama categories was 0% in Category 1 (insufficient), 1.92% in Category 2 (benign), 33.3% in Category 3 (atypical), and 100% in both Category 4 (suspicious for malignancy) and Category 5 (malignant). When malignant cases were considered positive, the diagnostic performance showed a sensitivity of 87.5%, specificity of 96.4%, PPV of 77.7%, and NPV of 98%.

Conclusion

The recategorization based on the IAC Yokohama System successfully classifies breast lesions according to their ROM and offers direction for the management strategy of each category. The adoption of the IAC Yokohama System for breast cytopathology reporting, with its standardized terminology, ensures improved diagnostic clarity, consistency, and accuracy, while also facilitating better prediction of the ROM and guiding clinical management and patient outcomes.

## Introduction

Breast cancer is a leading health concern among women due to its high mortality and morbidity rates. Recent GLOBOCAN 2018 data produced by the International Agency for Research on Cancer (IARC) from 185 countries reported 2.3 million new cases of breast cancer and a mortality rate of 6.9% [1,2].

Breast cancer has been ranked as the number one cancer among Indian females, with a mortality rate of 12.7 per 100,000 women [3]. Studies conducted in India and around the world have shown a marked rise in the incidence, morbidity, and cancer-related deaths on the Indian subcontinent [4,5].

A significant proportion of surgical cases involve breast diseases in both developed and developing countries; hence, the need arises to distinguish benign from malignant lesions prior to definitive treatment. With increasing awareness of breast cancer, there has been a parallel rise in anxiety and psychological stress among women, many of whom tend to interpret any breast-related symptom as suggestive of malignancy [6].

Fine-needle aspiration cytology (FNAC) is a minimally invasive, rapid, and cost-effective diagnostic procedure that is simple to perform and associated with minimal patient discomfort. It has been found to be a very reliable method for diagnosing breast lesions prior to surgery. It is considered the quickest and most reliable method for diagnosing breast lesions [7,8,9].

We use the terminology of the IAC Yokohama System for Reporting Breast Fine Needle Aspiration Cytopathology (IAC Yokohama System) to report cytology of all palpable breast lumps [10,11].

The system is designed to establish comprehensive best practice guidelines covering the indications and technical aspects of breast fine-needle aspiration biopsy (FNAB), including smear preparation [12]. Furthermore, it aims to develop structured checklists of essential cytological features to assist in the accurate diagnosis of specific breast lesions and to improve the overall interpretation of breast cytology smears [13].

Cytological grading of breast carcinoma was performed according to Robinson’s criteria, which incorporate six cytological parameters: cell dissociation, nuclear size, cell uniformity, nucleoli, nuclear margins, and chromatin pattern. Each parameter is scored on a scale of 1 to 3, and the aggregate score is used to derive the final cytological grade. Scores between 6 and 11 were assigned a grade of 1, scores between 12 and 14 were assigned a grade of 2, and scores between 15 and 18 were assigned a grade of 3 [14,15,16].

The IAC Yokohama System is employed to evaluate diagnostic and prognostic ancillary techniques and to offer evidence-based recommendations for their application [17,18]. Furthermore, it promotes standardized reporting by introducing well-defined categorical terminology, thereby ensuring international reproducibility and providing a structured format for reporting breast cytology findings [19].

Additionally, the integration of well-defined diagnostic categories with corresponding management recommendations and options strengthens training and quality assurance measures, thereby facilitating wider acceptance and utilization of the system among clinicians.

It includes five diagnostic categories: insufficient/inadequate (code 1), benign (code 2), atypical (code 3), suspicious of malignancy (code 4), and malignant (code 5). The risk of malignancy (ROM) for each category is calculated with the exact 95% binomial CIs [20,21,22]. This study was conducted to determine the profile of breast lesions on FNAC, classify them into the five diagnostic categories, and determine the diagnostic performance of FNAC, including its sensitivity and specificity in distinguishing benign from malignant lesions, through correlation with corresponding histopathological findings.

## Materials and methods

This study was conducted in the Department of Pathology, Indira Gandhi Medical College and Research Institute, Puducherry, using retrospective consecutive cases over a period of three years, after approval from the IRC and IEC. Consecutive cases of breast FNAC with histopathological follow-up were included in the study. Cases with no histopathological report, inadequate clinical details, or inadequate follow-up were excluded. A total of 63 cases were included. The FNAC details were retrieved from the investigation forms. The clinical and imaging details were collected from the FNAC investigation forms and histopathology request forms. The cytology slides of all breast lesions were retrieved from the filing section, and cytology grading was performed by two independent pathologists using the IAC Yokohama classification into five diagnostic categories: insufficient/inadequate (code 1), benign (code 2), atypical (code 3), suspicious of malignancy (code 4), and malignant (code 5). The results were further compared with the histopathology reports in order to validate the diagnostic importance of the Yokohama classification.

Statistical analysis 

Data entry was done using an MS Excel sheet and analyzed using SPSS software version 20. The FNAC cases were categorized into five categories as per the IAC Yokohama classification and correlated with the histopathology reports. The ROM was calculated for each category as the number of malignant cases confirmed histologically. Using the histological diagnosis as the gold standard, sensitivity, specificity, positive predictive value (PPV), and negative predictive value (NPV) were calculated.

All procedures performed in the current study were approved by the Institute Research and Ethics Committee (reference number No. 537/IEC-38/PP-35/2023, dated 05.09.2023) in accordance with the revised 2024 Declaration of Helsinki and its later amendments. Formal written informed consent was not required, as a waiver was granted by the appropriate Institute Research Ethics Committee.

## Results

**Figure 1:**
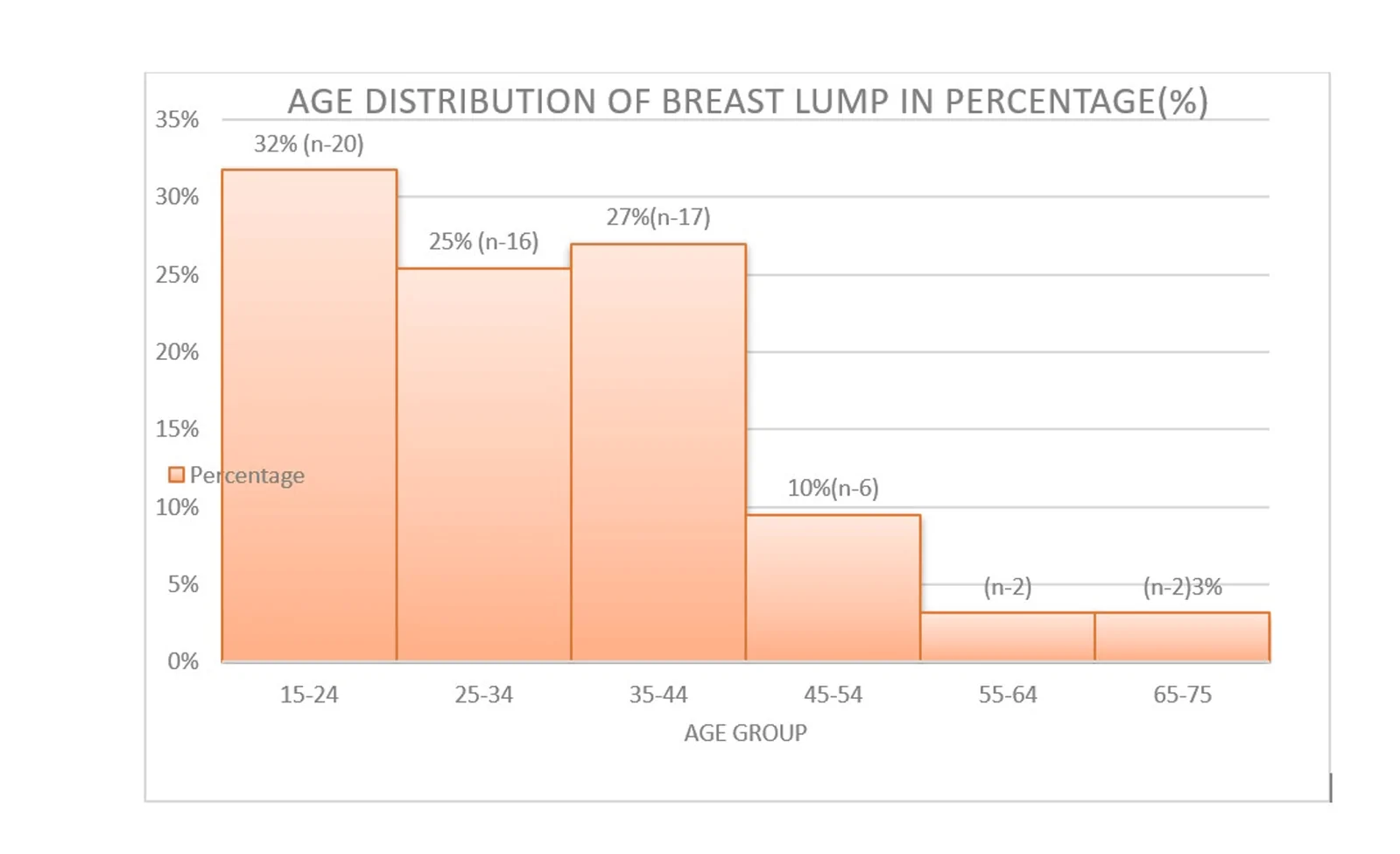
Bar chart showing the percentage distribution of breast lump cases across different age groups. The highest proportion of cases was observed in the 15-24 years age group (32%), followed by the 35-44 years (27%) and 25-34 years (25%) groups. A marked decline in frequency was seen in older age groups, with 10% of cases in the 45-54 years category and only 3% each in the 55-64 and 65-74 years groups.

The study comprised 63 patients with breast lesions who underwent FNAC and whose respective histopathology reports were available. The age-wise distribution of patients presenting with breast lumps showed that the majority of cases occurred in the younger and middle age groups. The highest proportion was observed in the 15-24 years age group (32%), followed by the 35-44 years age group (27%) and the 25-34 years age group (25%). A comparatively lower percentage was noted in the 45-54 years age group (10%), as shown in Figure 1.

The prevalence further declined in the older age groups, with only 3% of cases each in the 55-64 years and 65-74 years categories. This pattern indicates that breast lumps were more commonly reported among younger women, emphasizing the need for increased awareness, early clinical evaluation, and screening practices in this population.

**Figure 2:**
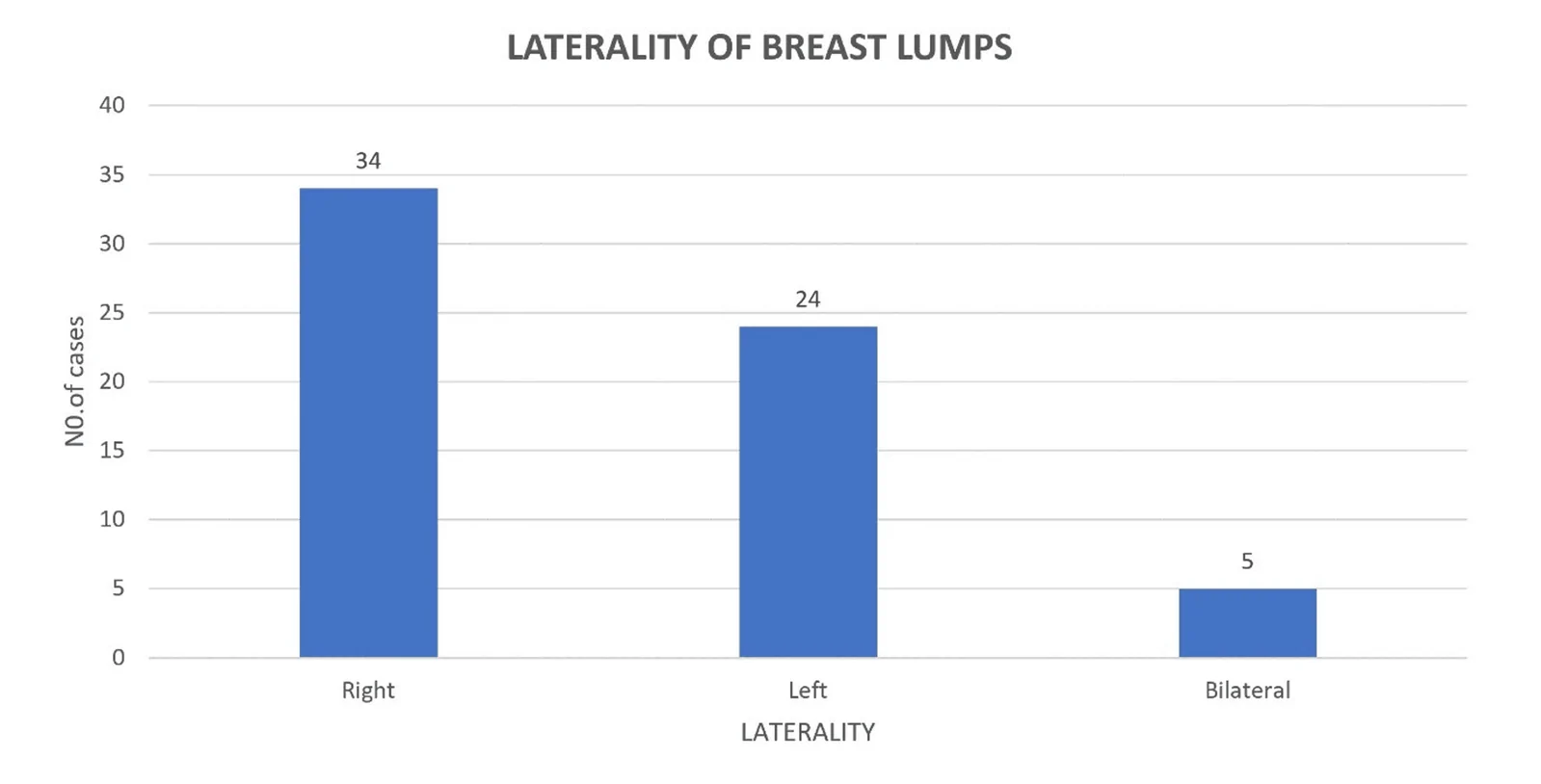
Bar chart illustrating the distribution of breast lumps according to laterality.

The distribution of breast lumps based on laterality revealed that the majority of cases involved the right breast. Out of the total study population, 34 cases were observed in the right breast, followed by 24 cases in the left breast. Bilateral breast lumps were comparatively less common, accounting for only 5 cases, as shown in Figure 2.

**Figure 3:**
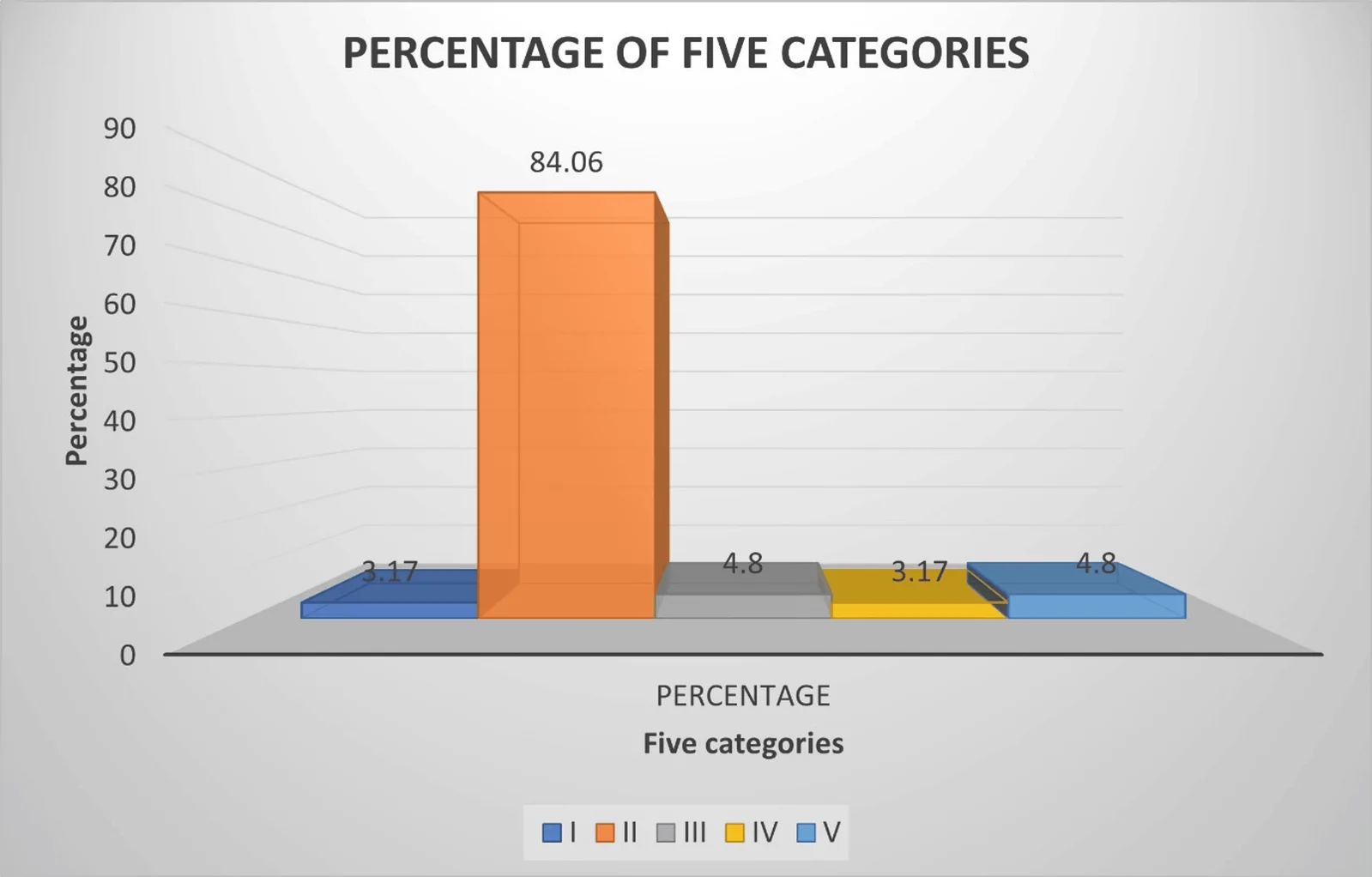
Bar chart depicting the percentage distribution of cases across the five IAC Yokohama categories. Category II constituted the vast majority of cases, accounting for 84.06%, while Categories III and V each represented 4.8% of cases. Categories I and IV were the least common, each comprising 3.17% of the total. Overall, the findings demonstrate a marked predominance of Category II lesions, with all other categories contributing only a small proportion to the total distribution. IAC: International Academy of Cytology.

On recategorizing breast cytology cases according to the International Academy of Cytology (IAC, 2016), Category I (insufficient material), Category II (benign), Category III (atypical), Category IV (suspicious), and Category V (malignant) comprised 3.17%, 84.06%, 4.8%, 3.17%, and 4.8% of cases, respectively, as shown in Figure 3.

**Figure 4:**
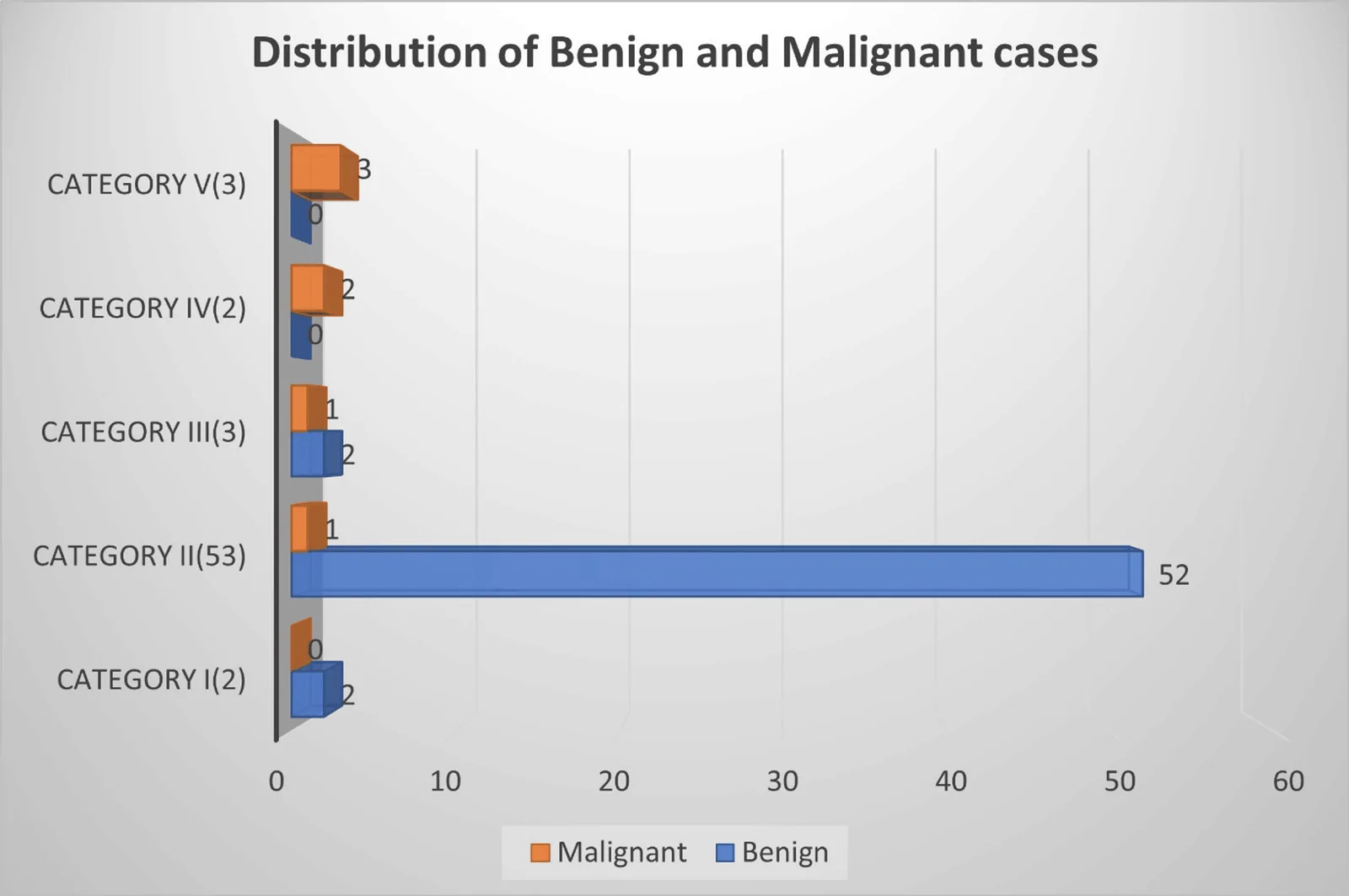
Bar chart illustrating the distribution of benign and malignant breast lesions across different diagnostic categories.

Among these 63 cases, two cases categorized under Category I (insufficient material) were diagnosed as fibroadenoma on histopathology. Out of 53 cases in Category II (benign), 52 cases were reported as fibroadenoma, fibrocystic disease, mastitis, abscess, and benign phyllodes, while one case was reported as malignant. Out of three cases in Category III (atypical), two cases were benign and one case was malignant. Two cases under Category IV (suspicious of malignancy) and three cases under Category V (malignant) were all reported as malignant (invasive ductal carcinoma), as shown in Figure 4.

**Figure 5:**
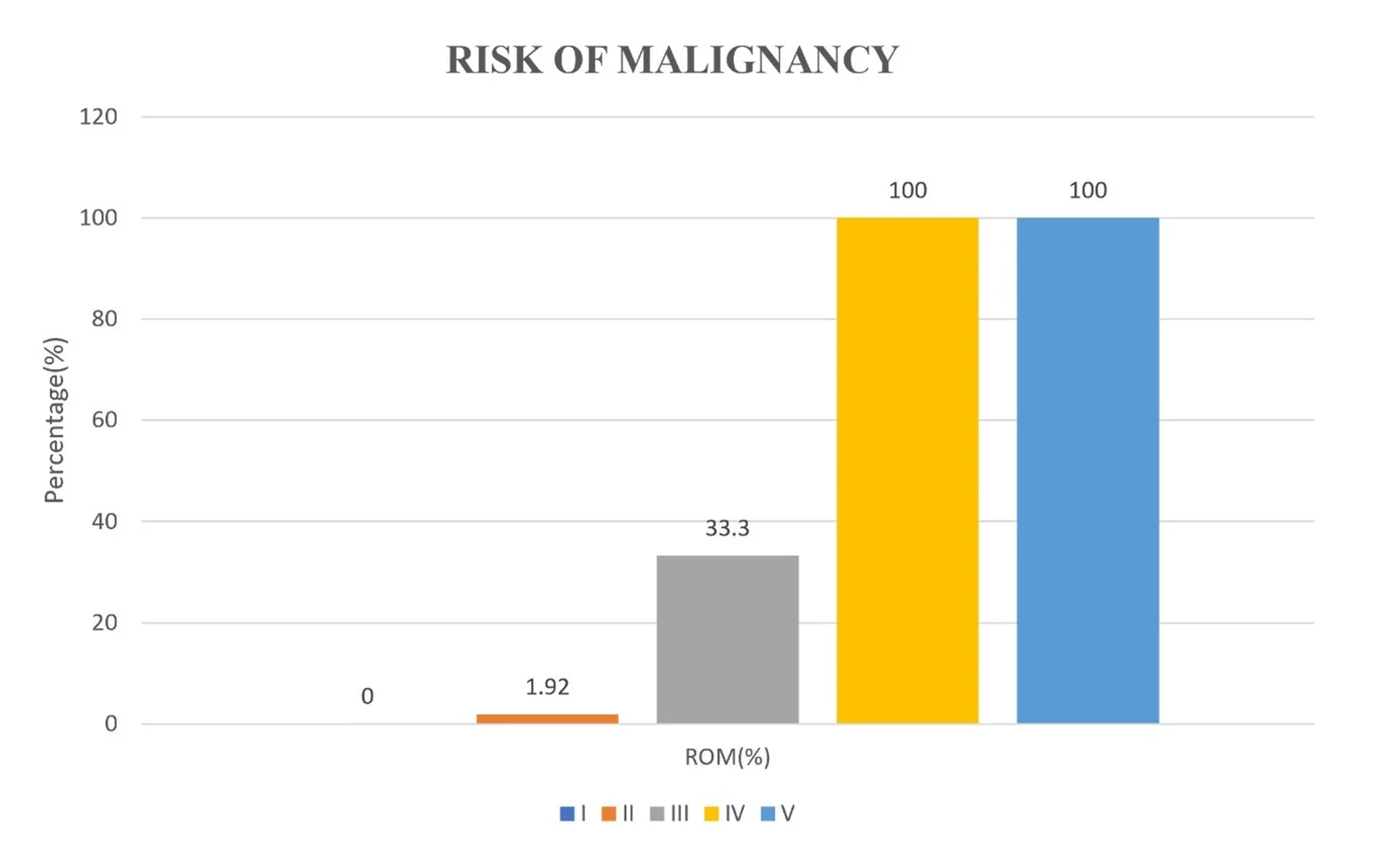
Risk of malignancy (ROM) associated with each diagnostic category in breast FNAC. Bars represent the percentage ROM for Category I (non-diagnostic/inadequate, 0%), Category II (benign, 1.92%), Category III (atypical, probably benign, 33%), Category IV (suspicious, probably malignant, 100%), and Category V (malignant, 100%). FNAC: Fine-needle aspiration cytology.

The ROM varied across the different diagnostic categories. It was 0% in Category I (insufficient), 1.92% in Category II (benign), 33.3% in Category III (atypical), and 100% in both Category IV (suspicious for malignancy) and Category V (malignant), as illustrated in Figure 5.

Considering histopathological examination as the gold standard for the diagnosis of breast lesions, FNAC showed a sensitivity of 87.5% and a specificity of 96.4%. The PPV and NPV were 77.7% and 98%, respectively, as presented in the Table 1.

**Table 1:** Diagnostic performance of the five-tier breast FNAC reporting system. Sensitivity, specificity, PPV, and NPV were calculated by considering Categories IV and V as “test positive” and Categories I-III as “test negative,” with histopathological diagnosis as the gold standard (n = 63). FNAC: Fine-needle aspiration cytology; PPV: Positive predictive value; NPV: Negative predictive value.

Sl. No.	Parameter	Percentage (%)
1	Sensitivity	87.50%
2	Specificity	96.40%
3	PPV	77.70%
4	NPV	98%

**Figure 6:**
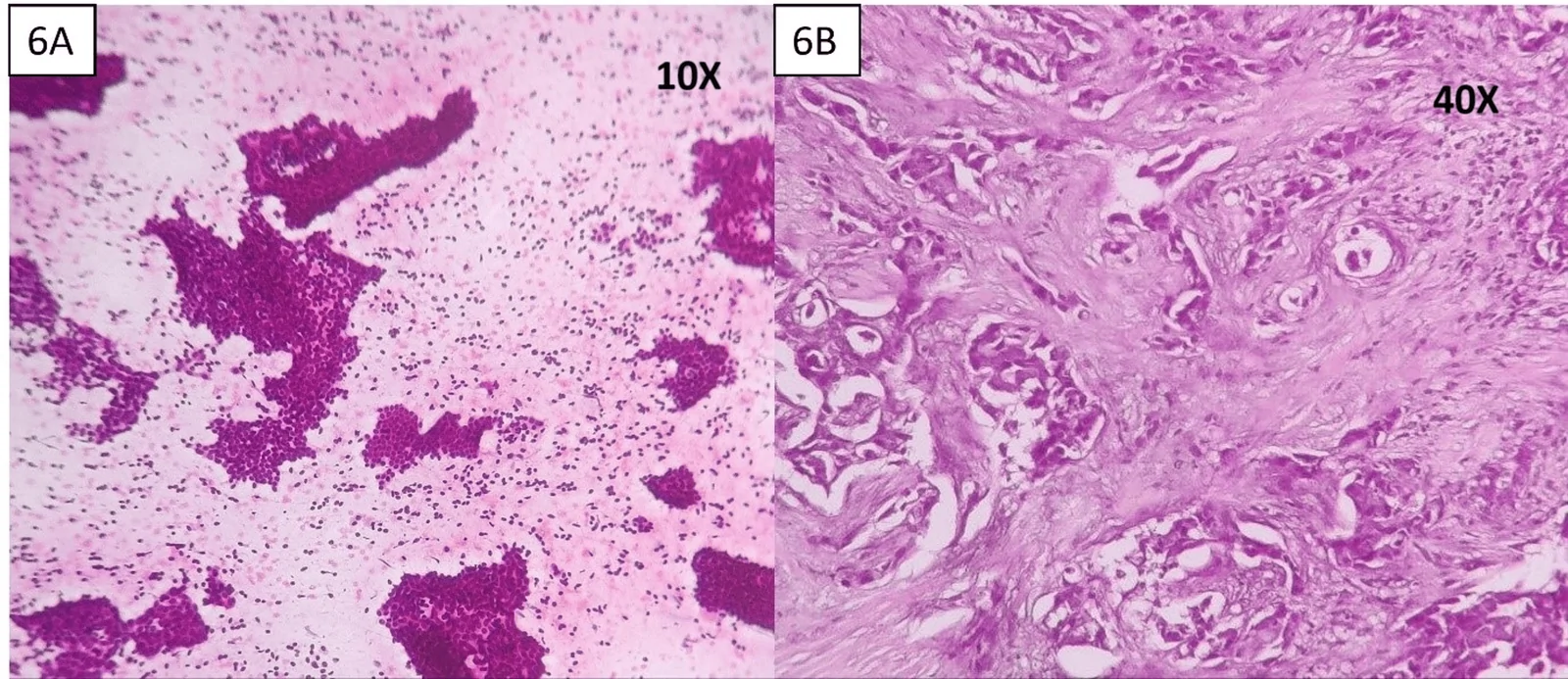
6A. Photomicrograph of cytology smear (H&E stain, ×10 magnification) showing features of fibroadenoma. Flat sheets of benign ductal epithelial cells with myoepithelial cells and bipolar naked nuclei are seen in a clean background (Category II: benign). 6B. Photomicrograph (H&E stain, ×40 magnification) showing the histological diagnosis of infiltrating ductal carcinoma, not otherwise specified (NOS). Note: The irregular infiltrative glands with malignant epithelial cells exhibiting nuclear pleomorphism, prominent nucleoli, and desmoplastic stromal reaction.

Figure 6 demonstrates a case of cytology-histopathology discordance. The FNAC smear (Figure 6A) showed features suggestive of fibroadenoma, including cohesive sheets and branching clusters of ductal epithelial cells with uniform nuclei, the presence of myoepithelial cells, and a relatively clean background, leading to a benign cytological interpretation. However, histopathological examination (Figure 6B) revealed invasive breast carcinoma characterized by infiltrating malignant glands, marked cellular atypia, stromal desmoplasia, and loss of normal ductal architecture.

**Figure 7:**
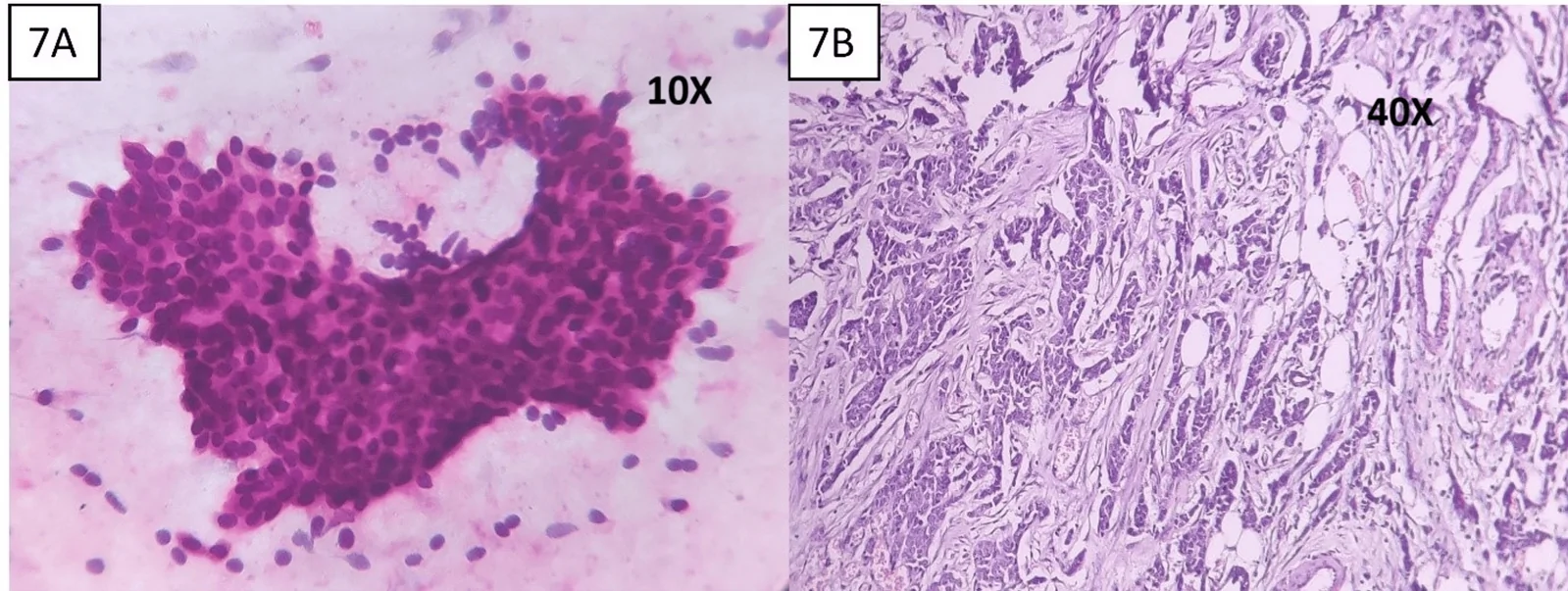
7A: Photomicrograph of cytology smear (H&E stain, ×10 magnification) showing features suspicious for malignancy (Category IV). Cohesive clusters of atypical epithelial cells with irregular nuclei, prominent nucleoli, and scant cytoplasm are seen in a necrotic background. 7B: Photomicrograph (H&E stain, ×40 magnification) showing the histological diagnosis of infiltrating ductal carcinoma, not otherwise specified (NOS). Tumor cells form angulated glands and single files infiltrating dense desmoplastic stroma with lymphocytic infiltrate.

Figure 7 demonstrates the cytological and corresponding histopathological findings in a case categorized as suspicious for malignancy. The FNAC smear (Figure 7A) showed highly cellular smears composed of loosely cohesive and discohesive clusters of atypical ductal epithelial cells. The cells exhibited an increased nuclear-cytoplasmic ratio, nuclear pleomorphism, hyperchromasia, and prominent nucleoli, leading to a cytological diagnosis of Category IV (suspicious for malignancy). Subsequent histopathological examination (Figure 7B) confirmed the diagnosis of invasive breast carcinoma of no special type (NST), characterized by invasive malignant ductal structures with marked cellular atypia and a desmoplastic stromal reaction.

**Figure 8:**
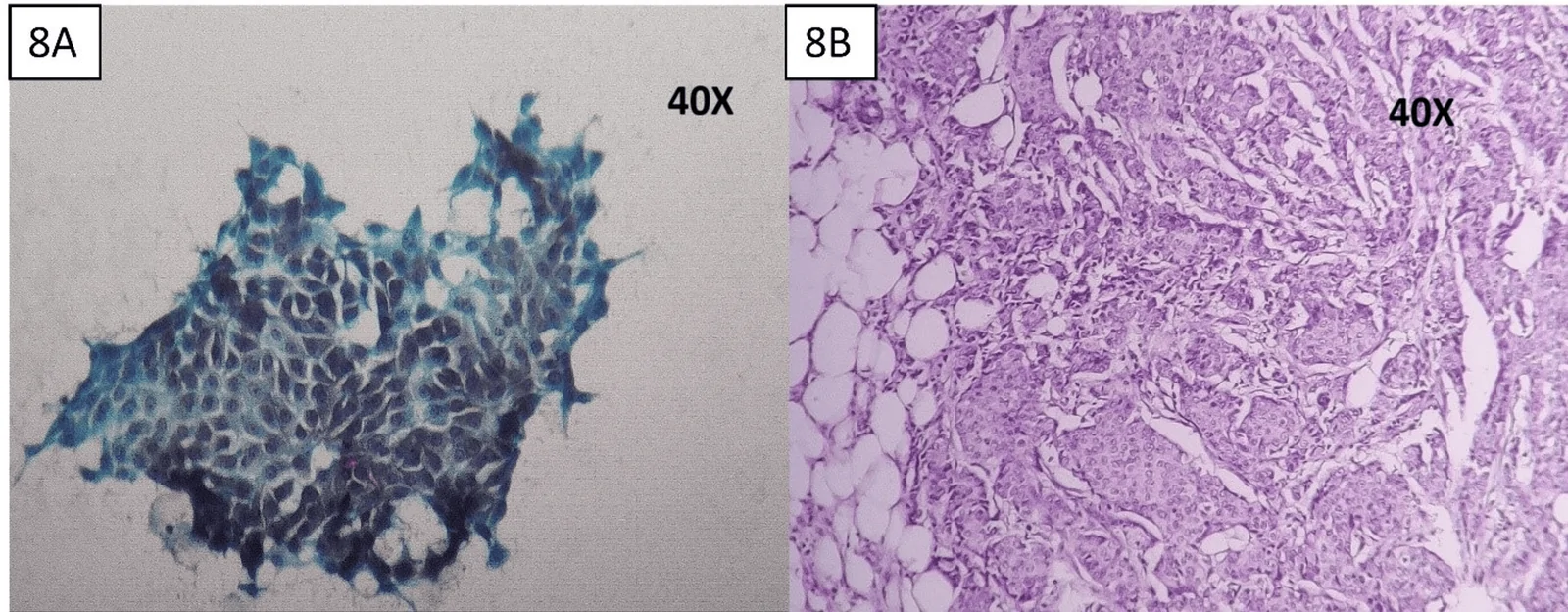
8A: Photomicrograph of cytology smear (Papanicolaou stain, ×40 magnification) showing features of malignancy (Category V). 3D malignant epithelial clusters with a high nuclear-to-cytoplasmic ratio, coarse chromatin, irregular nuclear membranes, prominent nucleoli, and tumor diathesis are seen. 8B: Photomicrograph (H&E stain, ×40 magnification) showing the histological diagnosis of infiltrating ductal carcinoma, not otherwise specified (NOS). Ill-formed glands and solid nests of atypical cells with marked pleomorphism and brisk mitotic activity are seen within a sclerotic stroma.

Figure 8 depicts the cytological and corresponding histopathological findings in a case diagnosed as malignant. The FNAC smear (Figure 8A) showed highly cellular smears composed of markedly atypical ductal epithelial cells arranged in irregular clusters and dispersed singly. The cells exhibited a high nuclear-cytoplasmic ratio, significant nuclear pleomorphism, hyperchromatic nuclei, irregular nuclear membranes, and prominent nucleoli, consistent with a Category V (malignant) diagnosis. Subsequent histopathological examination (Figure 8B) confirmed the diagnosis of invasive breast carcinoma of NST, characterized by infiltrating malignant ductal structures, marked cytological atypia, and stromal desmoplasia. This case highlights the high diagnostic accuracy of FNAC in identifying malignant breast lesions and reinforces its role as a reliable, rapid, and minimally invasive diagnostic tool in the evaluation of breast lumps.

## Discussion

Breast lumps constitute a common clinical presentation and may represent a wide spectrum of benign and malignant lesions. Accurate preoperative diagnosis is essential for appropriate management and to avoid unnecessary surgical interventions. The concept of triple assessment, which includes clinical examination, radiological evaluation, and pathological diagnosis, plays a pivotal role in the early detection and treatment of breast lesions. Among the pathological modalities, FNAC remains a simple, minimally invasive, cost-effective, and rapid diagnostic technique. However, the lack of uniform reporting in the past often led to variability in interpretation and communication between cytopathologists and clinicians. The introduction of the IAC Yokohama System has addressed these issues by providing standardized diagnostic categories with an associated ROM and management recommendations.

In the present study, the majority of patients belonged to the younger and middle age groups, with the highest frequency observed in the 15-24 years and 25-44 years age groups. This finding is consistent with the known epidemiological pattern in developing countries, where benign breast lesions such as fibroadenoma are more prevalent among younger women. Similar observations were reported by Chauhan V et al. [23], where the most commonly affected age group was 21-40 years. The relatively lower number of cases in older age groups in our study may reflect increased awareness and early consultation among younger patients.

The present study demonstrated a predominance of benign lesions, with Category II constituting the majority of cases. Fibroadenoma was the most common benign lesion, followed by fibrocystic changes and inflammatory conditions. This distribution is comparable to several previous studies, which have also reported a higher frequency of benign breast lesions on FNAC. The relatively low proportion of malignant cases in our study could be attributed to early presentation and increased screening practices.

The ROM in our study showed a progressive increase across the Yokohama categories, which validates the clinical utility of this classification system. Category I showed no ROM, while Category II demonstrated a low ROM of 1.92%, highlighting the reliability of benign cytological interpretation. Category III exhibited an intermediate ROM of 33.3%, reflecting the heterogeneous nature of atypical lesions and emphasizing the need for close clinical and radiological follow-up. Categories IV and V showed 100% ROM, indicating a strong correlation between cytological suspicion and histopathological confirmation of malignancy. These findings are in agreement with the study by Agarwal A et al., which also reported 0% ROM in Category I and 100% ROM in Category V [11]. However, the study by De Rosa F et al. reported a higher ROM in Category I, which could be due to sampling variability or differences in study design and population [10].

The sensitivity, specificity, PPV, and NPV of FNAC in the present study were 87.5%, 96.4%, 77.7%, and 98%, respectively. These results are comparable with those reported by Yalavarthi S et al. [24] and Mehra K et al. [25], although slight variations may be attributed to sample size, patient selection, and institutional experience. The high specificity and negative predictive value observed in our study indicate that FNAC is particularly effective in ruling out malignancy, thereby reducing unnecessary surgical biopsies. The comparatively lower positive predictive value may be related to the small number of malignant cases and occasional false-negative interpretations.

The discordant case observed in the present study, where a lesion diagnosed as fibroadenoma on cytology was confirmed as invasive carcinoma on histopathology, highlights the limitations of FNAC. Possible reasons include sampling error, tumor heterogeneity, or well-differentiated carcinoma mimicking benign lesions. Such cases underscore the importance of clinicoradiological correlation and repeat biopsy in suspicious or discordant cases.

The Yokohama System also improves communication between clinicians and pathologists by providing a clear framework for reporting and management. Category-wise ROM helps guide treatment decisions, such as observation for benign lesions, further evaluation for atypical lesions, and definitive management for suspicious or malignant lesions. The system also promotes quality assurance, training, and research by enabling uniform data comparison across institutions. Overall, the findings of the present study support the reliability and clinical relevance of the IAC Yokohama System in categorizing breast FNAC. The system provides standardized reporting, improves diagnostic accuracy, and assists in predicting the ROM, thereby facilitating appropriate patient management.

Furthermore, Birundha B et al. [26] observed that lower-grade tumours were more frequently hormone receptor positive, indicating a better prognosis and response to hormonal therapy. Early detection of such lesions through FNAC and standardized reporting systems like the Yokohama classification can facilitate timely histopathological confirmation and receptor testing. This multidisciplinary approach contributes to personalized management and improved survival outcomes in breast cancer patients. Therefore, integrating cytological findings with histological grading and receptor status enhances the overall diagnostic and therapeutic strategy in breast tumour management.

Limitations

The study included only 63 cases, which may limit the generalizability of the findings. Being conducted in a single tertiary care institution, the results may not reflect the broader population. Only cases with available histopathology were included, which may have led to selection bias. Although triple assessment is recommended, detailed radiological correlation was not statistically evaluated.

## Conclusions

The present study validates the clinical utility and diagnostic accuracy of the IAC Yokohama System for reporting breast FNAC. The system provides a structured, standardized, and reproducible approach for categorizing breast lesions with an associated risk of malignancy. The progressive increase in the risk of malignancy from Category I to Category V observed in this study confirms the reliability and predictive value of the Yokohama classification. The high specificity and NPV further highlight the usefulness of FNAC as a reliable method for ruling out malignancy and reducing unnecessary surgical interventions in benign breast lesions.

The findings also emphasize the importance of careful evaluation and follow-up of atypical and suspicious categories, along with clinicoradiological correlation and histopathological confirmation, particularly in discordant cases.

Future large-scale, multicentric, and prospective studies incorporating detailed clinicoradiological correlation are required to strengthen the validation of this system. The integration of ancillary techniques, such as immunocytochemistry and molecular markers, may further improve diagnostic accuracy, particularly in indeterminate categories.

FNAC remains a rapid, minimally invasive, cost-effective, and accurate diagnostic modality, especially in resource-limited settings.
